# Prediction of success of slings in female stress incontinence, statistical and AI modeling

**DOI:** 10.1038/s41598-025-12826-6

**Published:** 2025-08-07

**Authors:** Bassem S. Wadie, Ahmed Abdelrasheed, Mohammed Taha, Ahmed Abdelrahman, Bassam Mohamed, Alaa Saber, Ahmed Badawi

**Affiliations:** 1 Urology and Nephrology Center, Mansoura University, Mansoura, Egypt; 2 Biomedical Engineering, Faculty of Engineering, Cairo University, Giza, Egypt

**Keywords:** Sling, Female, Incontinence, Prediction, AI. SVM, ANN, Biotechnology, Urology

## Abstract

Studies on predicting the outcome of sling surgery are limited. Most depend on analysis of multiple confounding factors using regression models. However, their prediction results are limited. In this study, we tested a statistical regression model and an AI model for the prediction of the outcome of mid-urethral sling. Data were collected from 151 patients who underwent MUS surgery in our center from 2002 to 2022 and confounding factors that affect the outcome of the surgery at a minimum of one year. The study was divided into two phases. Phase I included the construction of a prediction model using binomial logistic regression. In phase II, we applied AI techniques (Artificial neural network (ANN) and Support Vector Machines (SVM) trying to obtain better predictions. Phase I: The logistic regression model predicted the outcome of surgery with overall accuracy of 90.7% and positive predictive value of 61.5% [X^2^ (11) = 46.24, P < 0.001]. Phase II: The data of the patients were entered as 10 features; 9 were predictors and the 10^th^ was the output. The output comprised 18 cases designated as ‘failure’ and 133 as ‘success’ output. The best model performance-wise was the (SVM) with 92% accuracy and 96% F1-score, which meets the industrial standards for predictive models. However, ANN produced 90% accuracy and 94% F1-score. However, our sample size is small. Prediction of the outcome of MUS surgery was achieved using different modalities with the best prediction of the outcome obtained by SVM method. This is helpful in future counseling of women undergoing sling surgery, whatever its type as to what to expect after surgery.

## Introduction

Stress urinary incontinence (SUI) is the most prevalent type of urinary incontinence (UI). Age, parity, and obesity were globally considered risk factors for the development of SUI. Other reports demonstrated factors such as hysterectomy, medical co morbidities, smoking, DM, and major depression^1–4^.

While pubovaginal sling (PVS) is the gold standard, the use of mid-urethral slings (MUS) became the most popular procedure since Ulmsten published his study on tension-free vaginal tape (TVT)^5,6^.

In addition, some studies demonstrated that primary and secondary outcomes of PVS and TVT were better than those of TOT^7,8^.

Different predictive models for prognostic and diagnostic purposes were created using "experience”. Logistic regression (LR) and artificial neural networks (ANN) are two examples. These models are based on 2 distinct yet similar fields; statistics and computer science^9^***.***

Compared to LR, ANN has various benefits: requiring less statistical training, detecting potential interactions between predictors, and complex nonlinear correlations. However, drawbacks include its black-box nature, the need for more complex computing procedures, the empirical nature of model construction, and the possibility of over-fitting the data, which could compromise the model’s ability to generalize^10^.

Support Vector Machines (SVM) is an algorithmic application of concepts from statistical learning, which helps constructing reliable estimators from data^11^. By resolving a constrained quadratic optimization problem, SVM construct the best separation boundaries between data sets. Different levels of nonlinearity and flexibility can be incorporated into the model by employing different kernel functions. SVM is developed from sophisticated statistical concepts and limitations on the generalization error can be eliminated^12,13^***.*** Our study aims at exploring different models which can help in predicting the outcome of MUS. We believe that the application of AI in this end is both timely and clinically relevant, as many studies^14–16^ failed to predict the success of MUS based on usual statistical methods.

## Materials and methods

All women underwent MUS in our facility from January 2002 to January 2020 with a minimum follow- up of 1 year, were retrospectively studied. *All methods were performed in accordance with the relevant guidelines and regulations.*

Inclusion and exclusion criteria were similar to previous report^7^. 257 patients were contacted by phone and asked to attend an outpatient clinic visit. Informed consent was obtained from all patients. The study design and protocol were approved by the local ethical/scientific committee of UNC. As the data were retrospective and no disclosure of patients’ identities were made, UNC IRB does not mandate informed consent forms to be collected.

Confounding factors were: age, body mass index (BMI), parity, previous pelvic surgery, pre-operative urodynamics (UDS). Follow up visits included per vaginal examination, stress test, pad test, post-void residual (PVR) and symptom scores.

The primary outcome is the construction of a prediction model that selects the patient with the best success rate. Cure is defined according to objective criteria (a negative stress test, a negative 1-h pad test and no retreatment) and subjective criteria (self-reported absence of symptoms, no leakage episodes). Failure was defined as persistent stress component.

Patients’ data were retrieved and reviewed regarding demographic data, surgical history, preoperative examination, pre-operative UDS, type of the sling, concomitant repair of prolapse, and BMI which was classified into values of more or less than 30. Parity was classified into values of more or less than 3. Abdominal leak point pressure (ALPP) was classified into 3 grades; > 90 (grade 1), 90—60 (grade 2) and < 60 (grade 3)^17^. Pre-operative bladder capacity was classified as normal or low (< 250 ml). The type of sling used was TVT, TOT or PVS.

Statistical analysis was carried out using SPSS version 24. In phase I, variables were categorical and Chi-Square test and McNemar’s test were used. Spearman’s correlation coefficient was used to define the association between variables. Binomial logistic regression analysis was used for the evaluation of the impact of independent variables on the prediction of the outcome of surgery.

Phase II included analysis of data through AI model (ANN and SVM). The output was divided into success or failure.

ANN consists of neurons in several layers, where each neuron is considered a mathematical unit. Each input is given a weight. During training, the weights and biases of all neurons get modified as the training continues with the target of reaching the best accuracy. Our model consists of 3 layers: input layer, a hidden layer, and an output layer. The first has 10 neurons (the number of input features). The hidden layer has 13 neurons and the output layer has 2 which correspond to the output classes 0 and 1.

The first 2 layers use the Rectified Linear Unit (ReLU) as the activation function while the output layer uses the Log-Softmax.

$$ReLU\left(x\right)=\text{max}\left(0, x\right)\text{log}\_\text{softmax}({x}_{j})=\text{log}\left(\frac{\mathit{exp}\left({x}_{j}\right)}{{\sum }_{i=0}^{c-1}\mathit{exp}\left({x}_{i}\right)}\right)\text{ where c is the number of classes}$$

In our study, we used the negative log-likelihood loss:

$$\text{negative}\_\text{log}\_\text{likelihood}\left(\text{X},\text{ c}\right)= -{X}_{c}$$

With this loss, we can calculate the gradients for each weight and bias of each neuron with a proper optimization function. We used the adaptive moment estimation (ADAM) optimizer, which modifies the weights and biases with the gradients calculated from the loss function. We trained the model for 50 epochs and used that number for quick redoing of iterations.

## Results

### Phase I

Analysis of data showed no statistical significance regarding different age groups or BMI groups. However, cases with BMI over 30 had the highest failure. Parity was statistically insignificant even though more failures were noted when parity was > 3. Women with previous pelvic surgery showed higher failure rate than those who had no pelvic surgery (P value < 0.05) (Table 1).

**Table 1:** The relationship of different covariates and the outcome of sling surgery at last follow up.

	Last Follow up		
	Success N (%)	Failure N (%)	Total N (%)
Age			
< 50	92 (89.3%)	11 (10.7%)	103 (100%)
> 50	41 (85.4%)	7 (14.6%)	48 (100%)
Total	133 (88.1%)	18 (11.9%)	151 (100%)
Pearson Chi-Square Value = 0.475 P = 0.491			
BMI			
< 30	41 (87.2%)	6 (12.8%)	47 (100%)
> 30	92 (88.5%)	12 (11.5%)	104 (100%)
Total	133 (88.1%)	18 (11.9%)	151 (100%)
Pearson Chi-Square Value = 0.046 P = 0.829			
Parity			
< 3	23 (92%)	2 (8%)	25 (100%)
> 3	110 (87.3%)	16 (12.7%)	126 (100%)
Total	133 (88.1%)	18 (11.9%)	151 (100%)
Pearson Chi-Square Value = 0.439 P = 0.508			
Uninhibited contractions			
Yes	12 (80%)	3 (20%)	15 (100%)
No	121 (89%)	15 (11%)	136 (100%)
Total	133 (88.1%)	18 (11.9%)	151 (100%)
Pearson Chi-Square Value = 1.035 P = 0.309			
Previous pelvic surgery			
Yes	42 (80.8%)	10 (19.2%)*	52 (100%)
No	91 (91.9%)	8 (8.1%)	99 (100%)
Total	133 (88.1%)	18 (11.9%)	151 (100%)
Pearson Chi-Square Value = 4.037 P < 0.05			
Abdominal leak point pressure			
> 90	37 (94.9%)	2 (5.1%)	39 (100%)
90 – 60	72 (90%)	8 (10%)	80 (100%)
< 60	24 (76.5%)	8 (23.5%)**	32 (100%)
Total	133 (88.1%)	18 (11.9%)	151 (100%)
Pearson Chi-Square Value = 7.209 P < 0.05			
Pre-operative bladder capacity			
Normal	117 (94.4%)	7*** (5.6%)	124 (100%)
Low	16 (59.3%)	11**** (40.7%)	27 (100%)
Total	133 (88.1%)	18 (11.9%)	151 (100%)
Pearson Chi-Square Value = 26.01 P < 0.001			
Concomitant repair of POP			
No	68 (90.7%)	7 (9.3%)	75 (100%)
Yes	65 (85.5%)	11 (14.5%)	76 (100%)
Total	133 (88.1%)	18 (11.9%)	151 (100%)
Pearson Chi-Square Value = 0.950 P = 0.330			
Type of sling			
TVT	49 (89.1%)	6 (10.9%)	55 (100%)
PVS	51 (96.2%)	2 (3.8%)	53 (100%)
TOT	33 (76.7%)	10 (23.3%)*****	43 (100%)
Total	133 (88.1%)	18 (11.9%)	151 (100%)
Pearson Chi-Square Value = 8.666 P < 0.05			

*Is a significant different group with Z score =  + 1.96 (Post Hoc Chi- Square test). **Is a significant different group with Z score =  + 2.1 (Post Hoc Chi- Square test. ***Is a significant different group with Z score =—2.0 (Post Hoc Chi- Square test. ****Is a significant different group with Z score =  + 4.3 (Post Hoc Chi- Square test. *****Is a significant different group with Z score =  + 2.2 (Post Hoc Chi- Square test.

Analysis of pre-operative UDS showed statistical significance regarding ALPP and pre-operative maximum bladder capacity. ALPP is thought to be a crucial predictor for the outcome of MUS. Patients with ALPP below 60 cmH2O showed higher failure rate than those with over 60 H2O (P < 0.05)**.** Also, low bladder capacity before surgery was accompanied with higher failure rate than those with normal capacity (P < 0.001)**.** A moderate correlation was reported between pre-operative bladder capacity and the outcome of surgery (*r* = 0.4).

Increased failure rate was noted in cases having TOT. (P < 0.05), while concomitant repair of POP, had no effect on the outcome of surgery. (Table 1).

#### Logistic regression

**Fig. 1:**
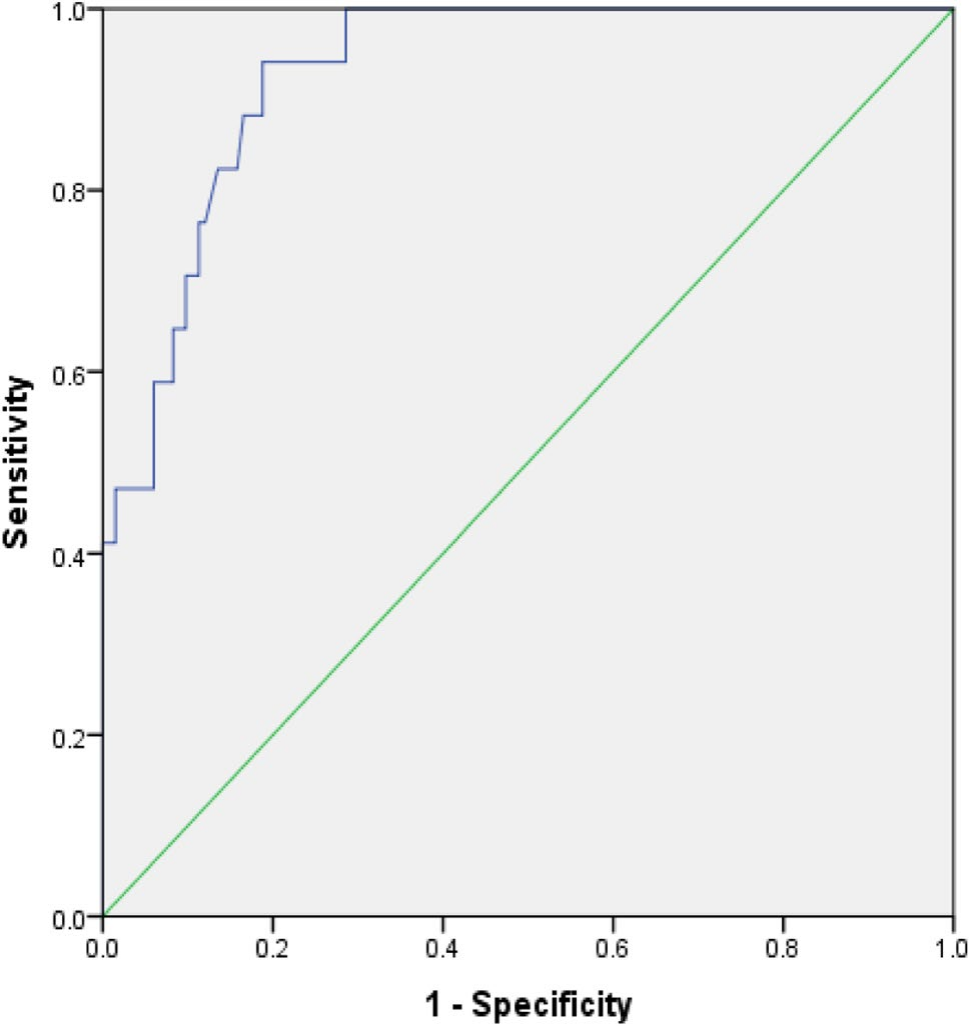
ROC curve for accuracy of LR model on the outcome of MUS.

Binomial logistic regression was performed including 151 patients in total, to assess the effects of age, BMI, parity, previous pelvic surgery (PPS), abdominal leak point pressure (ALPP), pre-operative maximum bladder capacity (Pre-Cap), uninhibited contraction (UC), concomitant repair of POP (CRPOP), and the type of the sling (independent variables) on the likelihood of success. The LR model was statistically significant in prediction of the results with an accuracy of 90.7% [X^2^ (11) = 46.24, P < 0.001]. According to the accuracy of this model, the area under the ROC curve was 0.93 (95% CI 0.883 to 0.977) (Fig. 1), which is important in discrimination according to Hosmer et al.^18^ with overall accuracy 90.7%, positive predictive value of 61.5% and negative predictive value of 93.4%.

**Fig. 2:**
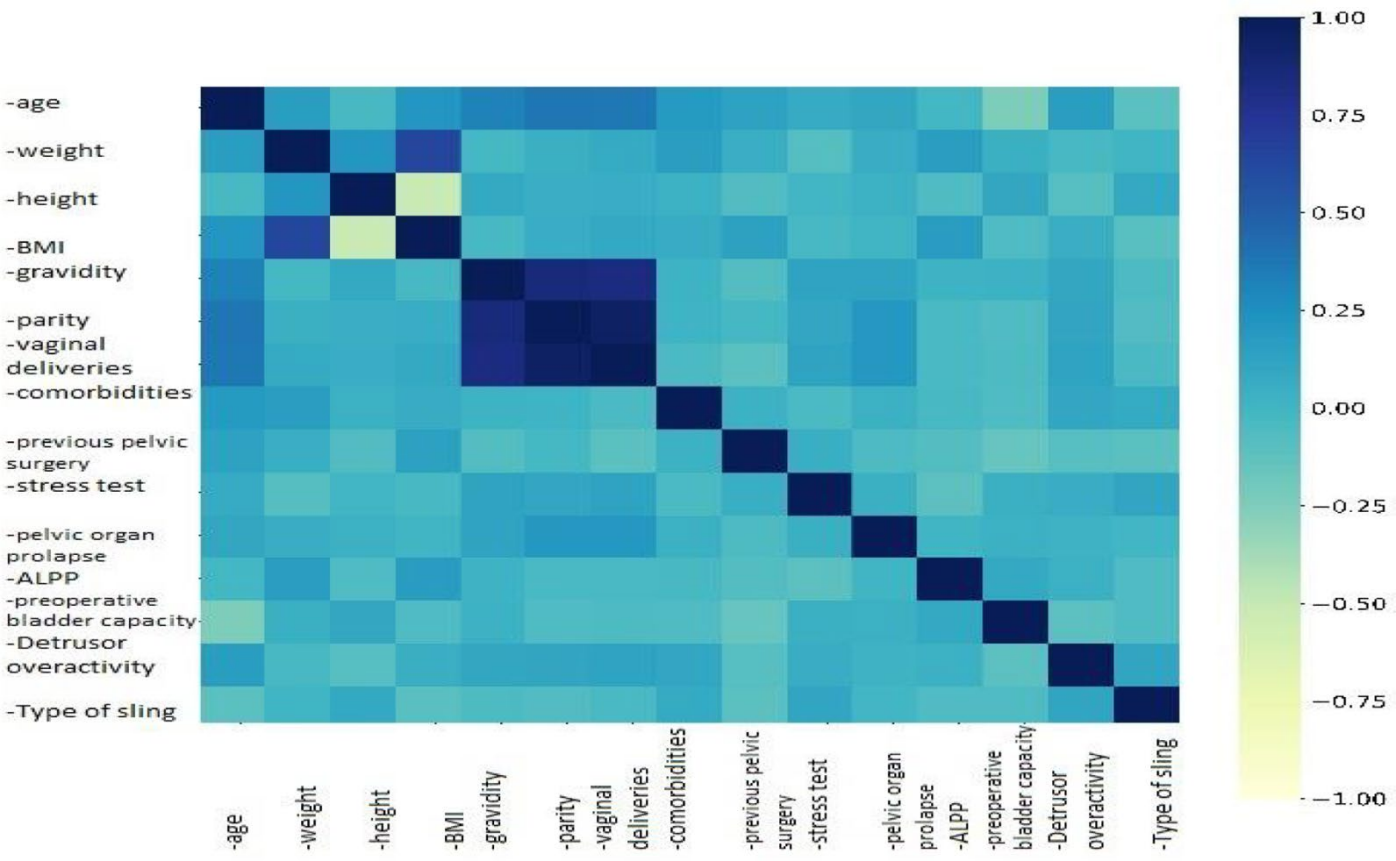
A heat map of the Spearman’s correlation coefficient between each pair of features.

Many predictive variables of this model were found statistically significant in the prediction of dependent variable as shown in Table 2, which demonstrates that: patients with previous pelvic surgery were 8 times more likely to have failure than those without, patients with < 60 ALPP had 24- and 6-times higher odds to have failure than patients with > 90 and 90–60 ALPP, respectively, patients with low pre-operative maximum bladder capacity had 29 times higher odds to fail surgery than those with normal capacity. Spearman’s correlation coefficient between bladder capacity and outcome of surgery was strong with *r* = 0.415 (P < 0.001) (Fig. 2) Patients with TOT sling type had 22 times higher odds to fail surgery than those with PVS.

**Table 2:** Logistic regression analysis of the likelihood of failure of the sling surgery.

Covariate	N	Odds Ratio	95% Confidence interval	P Value
Age				
> 50	48	(Ref)	(Ref)	(Ref)
< 50	103	0.887	(0.196 – 4.022)	0.877
BMI				
> 30	104	(Ref)	(Ref)	(Ref)
< 30	47	2.513	(0.572 – 11.038)	0.222
Parity				
> 3	126	(Ref)	(Ref)	(Ref)
< 3	25	0.588	(0.071 – 4.863)	0.622
PPS				
No	99	(Ref)	(Ref)	(Ref)
Yes	52	7.847	(1.642 – 37.504)	< 0.05
ALPP				
< 60	32	(Ref)	(Ref)	(Ref)
90 – 60	80	0.168	(0.036 – 0.789)	< 0.05
> 90	39	0.041	(0.002 – 0.879)	< 0.05
Pre-Cap				
Low	27	(Ref)	(Ref)	(Ref)
Normal	124	0.035	(0.007 – 0.172)	< 0.001
UC				
No	136	(Ref)	(Ref)	(Ref)
Yes	15	0.905	(0.135 – 6.073)	0.918
CRPOP				
Yes	76	(Ref)	(Ref)	(Ref)
No	75	0.458	(0.112 – 1.867)	0.276
Type of sling				
TOT	43	(Ref)	(Ref)	(Ref)
TVT	55	0.226	(0.048 – 1.061)	0.059
PVS	53	0.045	(0.004 – 0.464)	< 0.01

BMI = Body mass index; PPS = previous pelvic surgery more than 6 months ago; ALPP = Abdominal leak point pressure; Pre- Cap = Pre-operative maximum bladder capacity; UC = Uninhibited contractions; CRPOP = Concomitant repair of Pelvic organ prolapse; TVT = Tension-free vaginal tape; TOT = Transobturator tape; PVS = Pubovaginal sling.

### Phase II

Despite the promising results of binominal LR model, we explored ANN in an attempt to reach more accurate predictability.

**Fig. 3:**
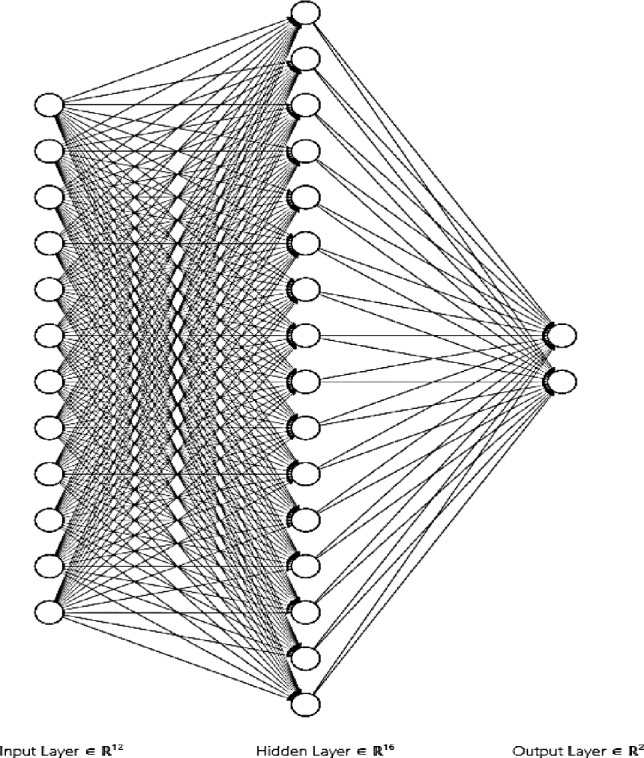
The structure of feed forward –back propagation ANN model.

The training set consisted of the records of 90 patients. After the network was trained accordingly, it predicted the outcome in a further 61 patients who comprised the testing set. The network was blinded to the output in the testing set. We evaluated the sensitivity and specificity of training as well as the testing sets using a confusion matrix. Figure 3 depicts the construction of the ANN.

**Fig. 4:**
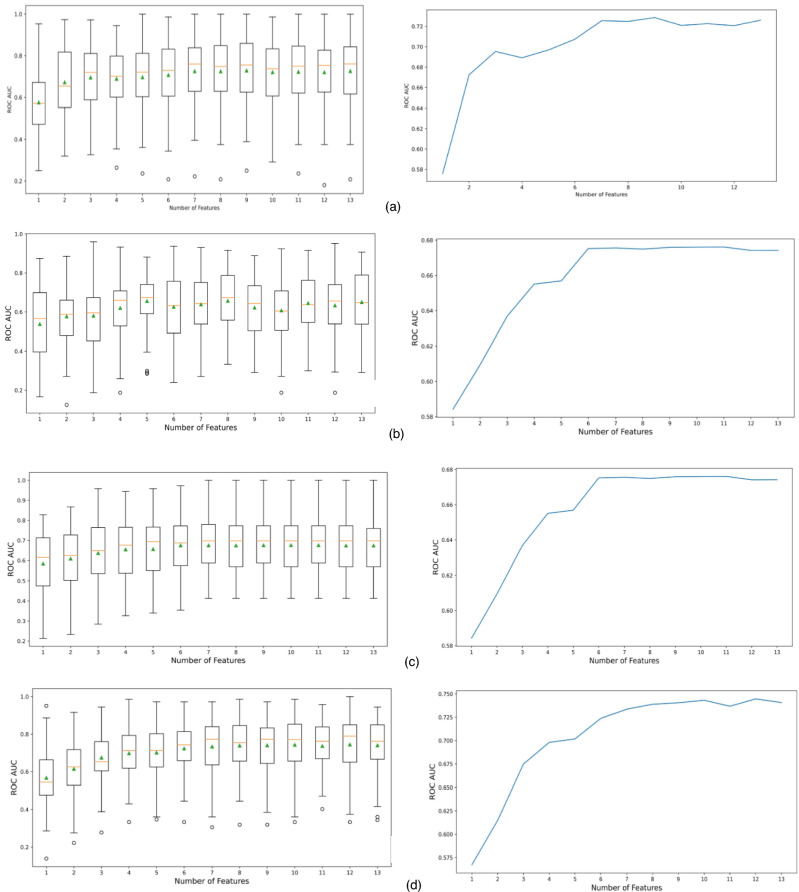
A box plot of the AUC of ROC scores and a plot of the mean of the scores resulted from a 10-repeats fivefold cross validation RFE for (**a**) random forest classifier, (**b**) decision tree classifier, (**c**) gradient boosting classifier, and (**d**) extra trees ensemble classifier.

Because of data imbalance and the relatively-small sample size, the neural network plateaued at 90.14% accuracy and F1-Score was 94.8%**.** Accuracy of ANN in discrimination between success and failure rendered an area under ROC curve of 0.7232. (Fig. 4).

**Fig. 5:**
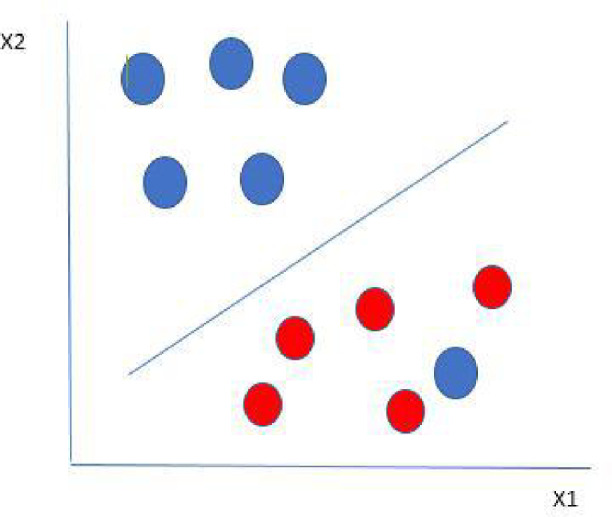
Graphic representation of SVM model.

The ANN model was not able to predict a single failure case. Therefore, we run our data via SVM and calculated accuracy, sensitivity, specificity, positive and negative predictive values. Figure 5 demonstrates graphic representation of SVM model.

The accuracy of the SVM model in the training set (using the records of 151 patients) was 93% accuracy and 96% F1-score. Table 3 shows the confusion matrix of SVM. In this model, accuracy is acceptable but its capability of prediction of failure is still unsatisfactory as positive predictive value did not exceed 40%.

**Table 3:** Confusion matrix of learning set of SVM.

State	Predicted failure	Predicted success
Failure	2	3
Success	1	55

## Discussion

In our study, cases were done only by 2 surgeons, accordingly the effect of the operator as a confounding factor could have been eliminated.

BMI ranged from 21.6 to 34.7 and despite of having statistically insignificant impact on the outcome. Our results were supported by the study of Bach et al.^19^ who reported low incidence of failure among patients with similar BMI range. Patients with BMI ranged from 35 to 50 reported higher incidence of failure and they should be offered weight loss first before surgery. This is going along with what Lee et al.^20^ have found in 138 women with SUI who underwent TVT and concluded that high BMI, low ALPP, and high grade of incontinence may impair the cure rate of the TVT.

Salhi et al. did a systematic review to evaluate the effect of previous pelvic surgery on the outcome of MUS. They conlcuded that one of the main predictive variables for adverse events following MUS was previous pelvic surgery [OR: 3.7 (CI 95%: 1.14–12.33); P = 0.029]^21^ and this is similar to what we found in our study where previous pelvic surgery is associated with higher failure rate [OR: 7.847 (CI 95%: 1.642–37.504); P < 0.05].

Nager et al. analyzed 260 cases of failure post MUS and reported that when ALPP is less than 86 cm H2O, risk of failure is 2-folds, regardless of the sling type [OR 2.23 (CI 95%: 1.20 – 4.14)]^22^ which is similar to what we reported in our study, when ALPP was less than 60 H2O, the chance of failure was 24- and 6-times higher odds to have failure than patients with > 90 and 90–60 ALPP, respectively [OR 0.041(CI 95%: 0.002– 0.879), p < 0.05].

In a landmark study including 565 women followed for 12-month, the rate of objectively-assessed success was 80.8% in the retropubic-sling group and 77.7% in the TOT group (3.0 percentage-point difference; 95% [CI], − 3.6 to 9.6)^23^. In our study, 151 patients had a success rate of 89.1% in the retropubic-sling group and 76.6% in the TOT group [OR :0.045 (CI 95%: 0.004 – 0.464), p < 0.01]***.***

Data on statistical models that predict the outcome of MUS are scarce. Through LR, four prediction models were applied to the Trial of Mid-urethral Slings (TOMUS) data set, to predict bothersome SUI, positive stress test, bothersome UUI, and adverse events within 12 months. The accuracy of these models reached 73% for discrimination between women who will or will not develop UI and 66% for prediction of adverse events^24,25^***.*** In our study, success was defined as absence of SUI during follow up. UUI was also evaluated and treated but not considered as failure. Our prediction model based on LR achieved an overall accuracy of 90.7%.

Our ANN is a back-propagation, feed forward type. Similar models were used in urology to diagnose and predict the prognosis of prostatic cancer^26,27^ achieving a positive predictive value of 94% for survival, and to form a reliable diagnostic tool based on symptoms and objective measurements^28^. However, the inclusion of subjective criteria might negatively affect the ability of the model to accurately predict failure and in future studies, a composite objective measured could be used. Besides, ANN has 2 main limitations: first its black-box nature and second it behaves better in larger sample size. As a machine learning tool for classification, SVM has gained popularity. It is straightforward and is considered as one type of data mining which is defined as obtaining valuable information from sizable data sets^29,30^^.^ SVM has the unique ability to perform linear classification using non-linear data; the so called “kernel trick”^31^.

However, SVM shortcomings include its sensitivity to parameter selection and high algorithmic complexity.

The wide application of AI models in the prediction of the outcome of sling surgery in women with SUI provides an efficient tool to counsel women prior to this surgery and give as best accurate prediction as we can get.

Also, SVM was used in oncology to predict 5-year overall survival after radical cystectomy^32^, post-cystectomy recurrence and survival^33^ and to differentiate angiomyolipoma from renal cell carcinoma based on texture analysis of CT images with 93.9% accuracy and 87.8% sensitivity^34^.

Conclusion: Building a statistical model regarding the outcome of MUS surgery with high accuracy and sensitivity is applicable through LR.

With an overall accuracy of 90.7% yet positive predictive value of 61.5%, LR model is far from optimum.

The use of AI is a better alternative to obtain valuable prediction of outcome of sling surgery. Our result using ANN was not much better than LR. Overall accuracy was 90.1% and the discrimination of failed cases was poor. SVM provided higher overall accuracy and predictive value of 93% and 96% respectively, mostly because of its kernel trick principle.

It is evident that using SVM, based on our results, is a way forward to better predict the outcome of sling surgery in the future.

Either one, a larger sample size is needed to obtain better prediction. We plan to further include more cases to our SVM model so that we can improve predictive outcome.

## Data Availability

Data of the study will be available upon request from the corresponding author.

## Abbreviations

AI: Artificial intelligence
ALPP: Abdominal leak point pressure
ANN: Artificial neural network
BMI: Body mass index
DM: Diabetes Mellitus
LR: Logistic regression
PVR: Post voiding residual urine
PVS: Pubovaginal sling
SUI: Stress urinary incontinence
SVM: Support vector machine
TOT: Transobturator tape
TVT: Tension free vaginal tape
UDS: Urodynamics
